# Deep convolutional neural networks for pan-specific peptide-MHC class I binding prediction

**DOI:** 10.1186/s12859-017-1997-x

**Published:** 2017-12-28

**Authors:** Youngmahn Han, Dongsup Kim

**Affiliations:** 1 Department of Bio and Brain Engineering, Korea Advanced Institute of Science and Technology, Daejeon, Republic of Korea; 2 Department of Convergence Technology Research, Korea Institute of Science and Technology Information, Daejeon, Republic of Korea

**Keywords:** T cell epitope prediction, Peptide-based vaccine development, Peptide-MHC class I binding prediction, Deep learning, Convolutional neural network

## Abstract

**Background:**

Computational scanning of peptide candidates that bind to a specific major histocompatibility complex (MHC) can speed up the peptide-based vaccine development process and therefore various methods are being actively developed. Recently, machine-learning-based methods have generated successful results by training large amounts of experimental data. However, many machine learning-based methods are generally less sensitive in recognizing locally-clustered interactions, which can synergistically stabilize peptide binding. Deep convolutional neural network (DCNN) is a deep learning method inspired by visual recognition process of animal brain and it is known to be able to capture meaningful local patterns from 2D images. Once the peptide-MHC interactions can be encoded into image-like array(ILA) data, DCNN can be employed to build a predictive model for peptide-MHC binding prediction. In this study, we demonstrated that DCNN is able to not only reliably predict peptide-MHC binding, but also sensitively detect locally-clustered interactions.

**Results:**

Nonapeptide-HLA-A and -B binding data were encoded into ILA data. A DCNN, as a pan-specific prediction model, was trained on the ILA data. The DCNN showed higher performance than other prediction tools for the latest benchmark datasets, which consist of 43 datasets for 15 HLA-A alleles and 25 datasets for 10 HLA-B alleles. In particular, the DCNN outperformed other tools for alleles belonging to the HLA-A3 supertype. The F1 scores of the DCNN were 0.86, 0.94, and 0.67 for HLA-A*31:01, HLA-A*03:01, and HLA-A*68:01 alleles, respectively, which were significantly higher than those of other tools. We found that the DCNN was able to recognize locally-clustered interactions that could synergistically stabilize peptide binding. We developed ConvMHC, a web server to provide user-friendly web interfaces for peptide-MHC class I binding predictions using the DCNN. ConvMHC web server can be accessible via http://jumong.kaist.ac.kr:8080/convmhc.

**Conclusions:**

We developed a novel method for peptide-HLA-I binding predictions using DCNN trained on ILA data that encode peptide binding data and demonstrated the reliable performance of the DCNN in nonapeptide binding predictions through the independent evaluation on the latest IEDB benchmark datasets. Our approaches can be applied to characterize locally-clustered patterns in molecular interactions, such as protein/DNA, protein/RNA, and drug/protein interactions.

**Electronic supplementary material:**

The online version of this article (10.1186/s12859-017-1997-x) contains supplementary material, which is available to authorized users.

## Background

Cytotoxic T lymphocytes (CTLs) play a key role in eliminating infections caused by intracellular pathogens. Since the CTL T-cell receptor recognizes foreign peptides in complex with major histocompatibility complex (MHC) molecules on the infected cell surface, the response of the host immune system to pathogens can be activated by peptide binding of MHC molecules. Determining peptides that bind specific MHC molecules is important for identifying T cell epitopes and can facilitate the development of peptide-based vaccines and design of immunotherapies. However, experimental identification of peptide-MHC is time-consuming and laborious; computer-assisted binding predictions can be a cost-effective and practical alternative and various methods have been developed [1].

Sette and Sidney grouped HLA class I (HLA-I) molecules into HLA supertypes using binding specificities characterized by the binding motifs of peptides [2]. Early peptide binding prediction methods were based on searching for allele-specific peptide binding motifs [3, 4]. As more experimental data became available, statistical methods have been developed using positional scoring matrixes that utilize amino acid occurrence frequencies at each position [5, 6]. Recently, more sophisticated machine learning methods [7–9] have generated the most successful results by training large amount of experimental data derived from public databases, such as the Immune Epitope Database [10]. Allele-specific machine learning methods generally achieve more accurate predictions as more data are learned for each HLA-I allele. A significant portion of currently available data was biased towards a limited number of common alleles [11], and this makes it difficult to predict peptide bindings for rare alleles. Sequence-based pan-specific methods have been proposed to overcome this problem and transfer the knowledge of other peptide-MHC binding information to improve the predictions for rare and even new alleles [12–14].

The pan-specific methods utilize information on not only the peptide sequence but also the MHC residues in peptide-MHC contact sites derived from the crystal structures of peptide-MHC complexes. The contact sites are clustered around the peptide anchor positions and the binding pockets of MHC molecules [14–16]. The amino acids of a peptide interact with MHC molecules in compensatory and synergistic manner rather than independently [17–19]. A large-scale structural simulation study of the peptide-MHC binding landscapes revealed statistically significant pairwise correlations in amino acid preferences at different positions of a peptide [15]. Many machine learning-based methods have a risk of learning the features associated with amino acids of peptide and the HLA-I molecule independently. Therefore, they could be less sensitive in recognizing the locally-clustered interactions, which could synergistically produce peptide-HLA-I binding.

Deep convolutional neural network (DCNN) is a branch of deep learning methods that extract and learn high-level representations (features or patterns) from the low-level raw data through nonlinear transformations of multiple layers. It was originally designed to process the spatial and temporal data, particularly two-dimensional images with multiple color channels. DCNNs are inspired by the animal visual cortex and imitate cognitive functions of the cortex using three key concepts: capturing local motifs of highly connected pixels, invariance to the motif location, and hierarchical composition of the local motifs [20]. DCNNs have achieved successful results in many object recognition and detection tasks [21–23]. Recent studies have proposed bioinformatics applications of DCNNs including protein contact predictions [24] and small molecule bioactivity predictions [25, 26].

In this study, we propose a novel method for pan-specific peptide-HLA-I binding prediction using DCNN. The peptide-HLA-I binding structure can be encoded into two-dimensional image-like array (ILA) data. A contact site between the peptide and MHC molecule is corresponded to a “pixel” of the ILA data. For each “pixel”, physicochemical property values of the amino acid pair at the contact site are assigned to its channels. The locally-clustered contact sites at peptide anchor positions and binding pockets of the HLA-I molecule form local motifs on the ILA data, which can be captured by DCNN. The resultant multi-channel ILA data were used to train the DCNN for peptide-HLA-I binding prediction. The DCNN showed a reliable performance for the independent benchmark datasets. In particular, we report that the DCNN significantly outperformed other tools in peptide binding predictions for alleles belonging to the HLA-A3 supertype. We also highlight the ability of DCNN to recognize the locally-clustered interactions in three peptides that bind to HLA-I molecules in synergistic manner.

## Methods

**Fig. 1:**
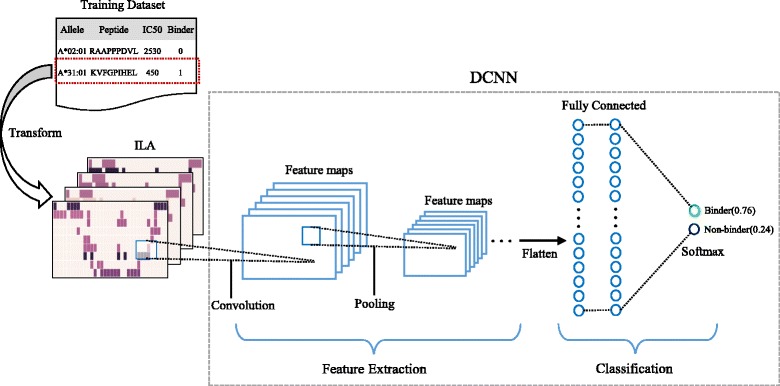
Schematic representation of overall training process of the DCNN. An ILA is converted from peptide binding information of training dataset. The DCNN extracts low-level features from the ILA and combines them into high-level features(motifs) through multiple convolutional and pooling layers. The DCNN learns these high-level features to be used for classifying the input ILA into binder or non-binder through fully connected layers

Figure 1 shows the schematic representation of overall training process of our DCNN. Each peptide binding information was encoded into ILA. The DCNN extracts low-level features from the ILA and combines them into high-level features(motifs) through multiple convolutional and pooling layers. The DCNN learns these high-level features to be used for classifying the ILA into binder or non-binder through fully connected layers.

### Training datasets

For benchmark with other tools, including NetMHCPan [14], SMM [5], ANN [7], and PickPocket [6], we used the same training dataset used in these tools. The dataset was compiled from three sources (the IEDB and the Sette and Buus laboratories) contained BD2009 and BD2013 data from [27] and additional binding data, which can be downloaded from the IEDB website (http://tools.iedb.org/mhci/download/). We used nonapeptide binding data for HLA-A and -B to generate a pan-specific prediction model. For the binary classification of peptide binding affinities, peptides with a half-maximal inhibitory concentration (IC50) value of less than 500 nM were designated as binders. In total, the training dataset consisted of 118,174 binding data covering 76 alleles: 37 HLA-A (72,551) and 39 HLA-B (45,623). Additional file 1: Table S1 shows the detailed description of the training dataset.

### Encoding peptide binding data into ILA data

**Fig. 2:**
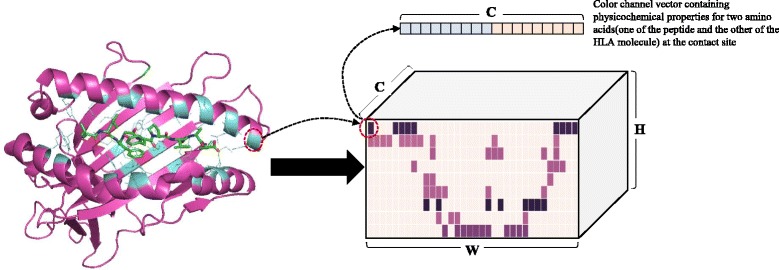
Encoding a peptide binding structure into an ILA. The left panel shows the nonapeptide (green)-HLA-A*02:01 (magenta) complex (PDB entry 1qsf). HLA residues at contact sites are depicted in cyan. The right panel shows the ILA data. The ILA width and height were the number of contact residue of the HLA molecule and the number of amino acids of the peptide, respectively. A contact site between the peptide and MHC molecule is corresponded to a “pixel” of the ILA. For each “pixel”, physicochemical property values of the amino acid pair at the contact site are assigned to its channels

As depicted in Fig. 2, a peptide binding structure can be encoded into a width (**W**) × height (**H**) ILA with **C** channels. The ILA width and height were the number of contact residue of the HLA molecule and the number of amino acids of the peptide, respectively. A contact site between the peptide and MHC molecule is corresponded to a “pixel” of the ILA. For each “pixel”, physicochemical property values of the amino acid pair at the contact site are assigned to its channels. We used 9 physicochemical scores out of 11 physicochemical scores suggested by [28] excluding two highly correlated scores (pairwise correlation, *R* ^*2*^ > 0.8) as the physicochemical property values of an amino acid; the channel size **C** is 18, the sum of the number of physiochemical scores of the amino acid pair at the contact site.

We used 34 HLA-I contact residues proposed by NetMHCPan [14]. Consequently, the nonapeptide-HLA-I binding data were encoded into ILA data with the dimension of 34 (width) × 9 (height) with 18 channels.

### Constructing and training the DCNN

**Fig. 3:**
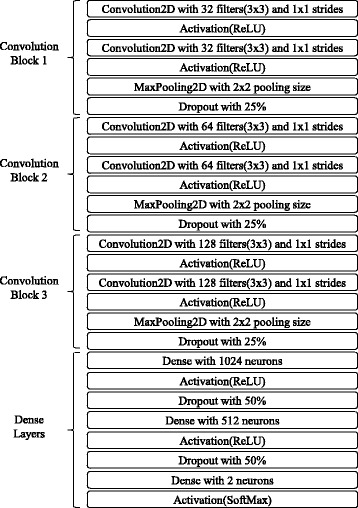
The DCNN architecture. The DCNN architecture is closely based on of the popular DCNN architecture proposed by Simonyan and Zisserman. Three convolution blocks with two convolution layers and a max pooling layer are concatenated, and three classification layers are then connected to the ends of the network. The dropout was next applied to each convolution block as a regularization. ReLU was used for the nonlinear transformation of the output value of each convolution layer

As shown in Fig. 3, our DCNN architecture is closely based on of the popular DCNN architecture proposed by Simonyan and Zisserma [23], which uses very small filters for capturing fine details of images and allows more elaborate data transformations through increased depth of the network. We concatenated three convolution blocks with two convolution layers and a max pooling layer, and then connected three dense layers to the ends of the network. In all convolution layers, convolution filters of 3 × 3 were used, and the numbers of filters for the convolution blocks were 32, 64, and 128, respectively. In order to avoid overfitting, we applied the dropout [29] acting as a regularization next to each convolution block. The ReLU [30] activation function was used for nonlinear transformation of the output value of each convolution layer. We used the Adam optimizer [31] with learning rate 0.001 for 200 epochs.

The DCNN was trained on the ILA data converted from 118,174 binding data covering 76 HLA-I alleles. In order to prevent the DCNN from overfitting the training data, the DCNN training was performed using leave-one-out and 5-fold cross-validations. The ILA data were split into 76 allele subsets in leave-one-out cross-validation and 5 equal sized subsets in 5-fold cross-validation, respectively. For each cross-validation round, a single subset was retained as the validation data for testing the DCNN, and the remaining subsets were used as training data. The cross-validation was repeated for the number of subsets: i.e., 76 times in leave-one-out cross-validation and 5 times in 5-fold cross-validation, with each subset used exactly once as the validation data. In a single cross-validation round, training-validation was repeated for maximum of 200 epochs. The training and validation losses were measured for each epoch, and the training process was stopped early at the epoch in which the validation loss had not been decreased for 15 consecutive epochs [32]. We implemented the DCNN using Keras library(https://github.com/fchollet/keras).

### Independent evaluation of the DCNN

Trolle et al. [33] developed a framework for automatically benchmarking the performance of peptide-MHC binding prediction tools. Based on this framework, the IEDB has evaluated the performance of participating prediction tools on IEDB experimental datasets, which are updated weekly, and published the results via the website (http://tools.iedb.org/auto_bench/mhci/weekly/). We performed a blind test of the DCNN using the latest experimental IEDB data accumulated since March 21, 2014. The accumulated data were grouped by IEDB references, alleles, and measurement types and split into 68 test subsets consisting of 43 subsets for 15 HLA-A alleles and 25 subsets for 10 HLA-B alleles (Additional file 2: Table S4). We performed the benchmark with other participating tools, including NetMHCPan, SMM, ANN, and PickPocket, for each subset. For the reliable benchmark, we used the latest standalone version of the prediction tools downloaded from the IEDB website (http://tools.iedb.org/mhci/download/), which were trained on the same training data as that of our DCNN. The F1 score, the harmonic mean of precision and recall, was used to quantify the prediction performance, where an F1 score reaches its best value at 1 and worst value at 0. The F1 score is defined as:

$$ F1=2\times \frac{precision\times recall}{precision+ recall}, $$

$$ precision=\frac{TP}{TP+ FP}, $$

$$ recall=\frac{TP}{TP+ FN}, $$

where TP, FP, and FN are the numbers of true positives, false positives, and false negatives, respectively.

### Identifying informative pixels recognized by the DCNN

In order to find locally-clustered interactions, informative pixels captured by the DCNN on the ILA classified as a binder were investigated. This was enabled due to the development of several recent methods that identify informative pixels of DCNN inputs, including Deconvnet [34], guided backpropagation [35], and DeepLIFT [36]. The informative pixels were found by using high-resolution DeepLIFT method in this study.

## Results and discussion

### Training results

In order to compare the prediction performance of the DCNN and other prediction methods, the DCNN was trained on the dataset that was used in other tools. The 118,174 nonapeptide-HLA-I binding data for 76 HLA-A alleles (72,551) and 37 HLA-B alleles (45,623) were encoded into the two-dimensional ILA data. The predictive performance was evaluated with leave-one-out and 5-fold cross-validation approaches. DCNN models were trained up to 200 epochs with early stopping condition. The mean validation losses were 0.318 in leave-one-out and 0.254 in 5-fold cross-validation, and the mean validation accuracies were 0.855 and 0.892, respectively (Table 1), and this indicate that our DCNN was able to be generally trained on the ILA data without much overfitting problems. Additional file 3: Table S2 and Additional file 4: Table S3 show the detailed cross-validation results.

**Table 1:** Summary of cross-validation results

	Average accuracy	Average loss
Leave-one-out	0.855	0.318
5-fold	0.892	0.254

### Independent evaluation of the DCNN

We performed a blind test of the DCNN using the latest IEDB experimental data accumulated since March 21, 2014. The data were grouped by IEDB references, alleles, and measurement types and split into 68 test subsets consisting of 43 subsets for 15 HLA-A alleles and 25 subsets for 10 HLA-B alleles. For each subset, the prediction performances of other prediction tools, including NetMHCPan, SMM, ANN, and PickPocket, were measured. The F1 scores were used to quantify their predictive performances. Table 2A and 2B summarize the prediction results for HLA-A and HLA-B test subsets, respectively, and Additional file 2: Table S4 shows the detailed prediction results. The mean and median of the F1 scores of the DCNN were 0.638 and 0.696, respectively; these values were slightly higher than those of other tools, suggesting that the DCNN was more reliable in nonapeptide-HLA-A binding predictions (Table 2A). The mean of the F1 scores of the DCNN was 0.593, which was almost the same as those of other tools; however, the median was 0.667, which was higher than that of the other tools, indicating that the DCNN was also reliable in nonapeptide-HLA-B binding predictions (Table 2B).

**Table 2:** Prediction results for HLA-I test subsets

(A) Summary of prediction results for 43 HLA-A test subsets					
	DCNN	NetMHCPan	SMM	ANN	PickPocket
Mean	0.638	0.608	0.601	0.579	0.561
Median	0.696	0.667	0.667	0.667	0.625
Standard Deviation	0.230	0.267	0.250	0.286	0.318
(B) Summary of prediction results for 25 HLA-B test subsets					
	DCNN	NetMHCPan	SMM	ANN	PickPocket
Mean	0.593	0.606	0.578	0.606	0.560
Median	0.667	0.625	0.615	0.643	0.593
Standard Deviation	0.286	0.286	0.302	0.290	0.277

In particular, our DCNN showed significantly higher prediction performance than other prediction tools for the subsets for HLA-A*31:01, HLA-A*03:01, and HLA-A*68:01 alleles belonging to the HLA-A3 supertype (Table 3).

**Table 3:** Prediction results for HLA-A*31:01, HLA-A*03:01, and HLA-A*68:01 alleles

IEDB ID	Allele	Meas. Type	DCNN	NetMHCPan	SMM	ANN	PickPocket
315312	HLA-A*31:01	binary	0.857	0.667	0.571	0.667	0.400
1031253	HLA-A*03:01	ic50	0.941	0.875	0.667	0.875	0.941
1026840	HLA-A*68:01	binary	0.340	0.275	0.456	0.208	0.045
1026840	HLA-A*68:01	ic50	0.667	0.600	0.583	0.444	0.143
	Mean		0.701	0.604	0.569	0.549	0.382

The HLA-A3 supertype were known to have important locally-clustered interactions that synergistically stabilizes the peptide-MHC complexes [26]. We thus investigated whether the trained DCNN was captured this features by inspecting its informative sites or pixels for three peptide-MHC complex pairs that were correctly predicted by our method but were failed in other methods: **KVFGPIHEL** for HLA-A*31:01, **RAAPPPPPR** for HLA-A*03:01, and **LPQWLSANR** for HLA-A*68:01.

**Fig. 4:**
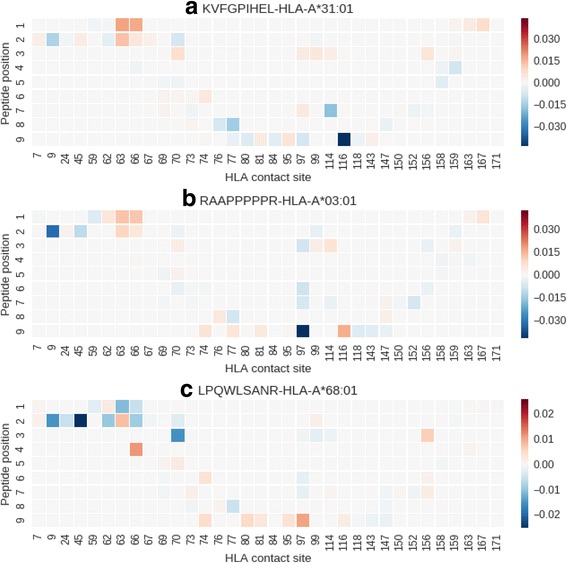
Informative pixels on the ILA data. (**a**) In KVFGPIHEL-HLA-A*31:01, the informative pixels with higher red intensities (red and blue intensities indicated the degree of contribution to the binder and non-binder, respectively) were dominant and locally-clustered at the positions 1, 2, and 3 (**b**) In RAAPPPPPR-HLA-A*03:01, informative pixels with higher red intensities were dominant and locally-clustered at the peptide positions 1 and 2 (**c**) In LPQWLSANR-HLA-A*68:01, informative pixels with red intensities were slightly dominant at positions 4, 5, and 6 and at the primary anchor position 9, with clustering at position 9

In **KVFGPIHEL**-HLA-A*31:01, the amino acids K, V, and F of the peptide were preferred at the primary and second anchor positions 1, 2, and 3, respectively, but the nonpolar and hydrophobic L was deleterious at the primary anchor position 9, and the charged H was tolerated at the secondary anchor position 7. We investigated the informative pixels on the transformed ILA data captured by the DCNN to identify the locally-clustered motifs at positions 1, 2, and 3. Fig. 4a shows that the informative pixels with higher red intensities (red and blue intensities indicated the degree of contribution to the binder and non-binder, respectively) were dominant and locally-clustered at the positions 1, 2, and 3, whereas the informative pixels with higher blue intensities were located at position 9. These findings were consistent with the fact that the locally-clustered patterns recognized by the DCNN were informative when the **KVFGPIHEL** was classified as a binder.

In **RAAPPPPPR**-HLA-A*03:01, the positively charged amino acid R of the peptide was preferred at the secondary anchor position 1, but the amino acids A, and R at the primary and secondary anchor positions 2, 3, and 9, respectively, were tolerated. Considering binding contributions of the individual amino acids at the primary and secondary anchor positions, the peptide could not be a binder. Fig. 4b shows that the informative pixels with higher red intensities were dominant and locally-clustered at the peptide positions 1 and 2, thus suggesting that the locally-clustered interactions between the amino acids at the peptide positions could produce stable binding together.

In **LPQWLSANR**-HLA-A*68:01, the positively charged R of the peptide was preferred at the primary anchor position 9, but the L, P, and Q were not preferred at the primary and secondary anchor positions 1, 2, and 3, respectively. The amino acids at positions 4, 5, 6, and 7 were tolerated. As shown in Fig. 4c, informative pixels with red intensities were slightly dominant at positions 4, 5, and 6 and at the primary anchor position 9, with clustering at position 9, thus indicating that amino acids at positions 4, 5, 6, and 9 synergistically induced stable binding.

We found that our DCNN was able to correctly predict the three binder peptides **KVFGPIHEL**, **RAAPPPPPR**, and **LPQWLSANR** with preferred amino acids only at some primary and secondary anchor positions but with amino acids that could synergistically induce stable binding. This small number of cases are insufficient to support the general higher prediction performance of DCNN approach for the HLA-A3 supertype, but these cases provide the possibilities that the DCNN can capture the locally-clustered interaction patterns in the peptide-HLA-A3 binding structures, which cannot be easily captured by other methods.

### Web server

**Fig. 5:**
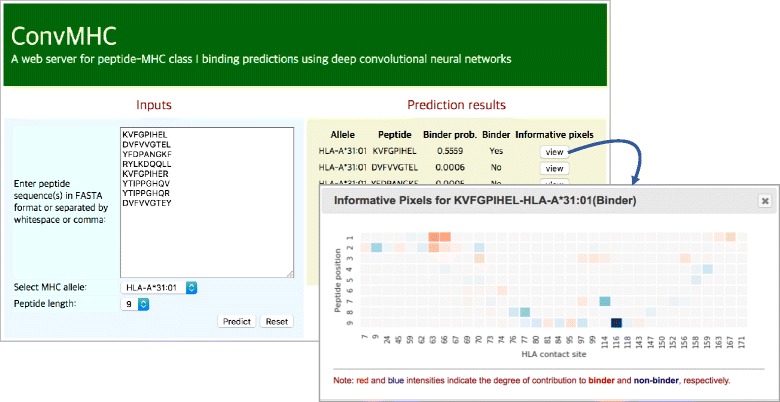
ConMHC Web Server. The main web interface of ConvMHC consists of the input form panel (left) and the result list panel (right). Users can submit multiple peptide sequences and a HLA-I allele in the input form panel. Once the prediction process is completed, the user can see the prediction results of the input peptides in the result list panel. For each prediction result, the user can also identify the informative pixels captured by the DCNN on the transformed binding ILA data through a pop-up panel

We developed ConvMHC(http://jumong.kaist.ac.kr:8080/convmhc), a web server to provide user-friendly web interfaces for peptide-MHC class I binding predictions using our DCNN. The main web interface consists of the input form panel (left) and the result list panel (right) as shown in Fig. 5. Users can submit multiple peptide sequences and a HLA-I allele in the input form panel. Once the prediction process is completed, the user can see the prediction results of the input peptides in the result list panel. For each prediction result, the user can also identify the informative pixels captured by the DCNN on the ILA data through a pop-up panel.

## Conclusions

In this study, we developed a novel method for pan-specific peptide-HLA-I binding prediction using DCNN trained on ILA data that were converted from experimental binding data and demonstrated the reliable performance of the DCNN in nonapeptide binding predictions through the independent evaluation on IEDB external datasets. In particular, the DCNN significantly outperformed other tools in peptide binding predictions for alleles belonging to the HLA-A3 supertype. By investigating the informative pixels captured by the DCNN on the ILA data converted from the binder nonapeptides that were predicted correctly by the DCNN but were failed in other methods, we found that the DCNN was better able to capture locally-clustered interactions that could synergistically produce stable binding in the peptide-HLA-A3 complexes: **KVFGPIHEL**-HLA-A*31:01, **RAAPPPPPR**-HLA-A*03:01, and **LPQWLSANR**-HLA-A*68:01.

We anticipate that our DCNN would become more reliable in peptide binding predictions for HLA-A3 alleles through further training and evaluations on more experimental data. DCNNs for MHC class II will be generated and evaluated in further studies. Moreover, our approaches described herein will be useful for identifying locally-clustered patterns in molecular binding structures, such as protein/DNA, protein/RNA, and drug/protein interactions. However, it is not easy to build a reliable prediction model using DCNNs because deep learning tasks require large amounts of training data to extract high-level and generalized representations from the data. Currently, in order to overcome the limited training data, state-of-the-art learning technologies, such as generative adversarial nets [37] and transfer learning [38] are attracting attentions. These technologies can be effectively applied to generate more reliable binding prediction models.

## Additional files

Additional file 1: Table S1. Detailed description of the training dataset. (XLSX 16 kb)

Additional file 2: Table S4. Detailed prediction results for the IEDB HLA-I benchmark datasets. (XLSX 18 kb)

Additional file 3: Table S2. Detailed results for leave-one-out cross-validation. (XLSX 16 kb)

Additional file 4: Table S3. Detailed results for 5-fold cross-validation. (XLSX 11 kb)

## Abbreviations

DCNN: Deep Convolutional Neural Network
HLA: Human Leukocyte Antigen, the human version of MHC
ILA: Image-Like Array
MHC: Major Histocompatibility Complex
